# P-253. The Impact of Armed Conflict on Advanced HIV Disease and Cryptococcal Meningitis Care in Northern Ethiopia

**DOI:** 10.1093/ofid/ofaf695.474

**Published:** 2026-01-11

**Authors:** Temesgen D Nurye, mengistu hagazi, Carla Y Kim

**Affiliations:** Columbia university, Addis Ababa, Adis Abeba, Ethiopia; Mekelle University, Mekelle, Tigray, Ethiopia; Columbia university, Addis Ababa, Adis Abeba, Ethiopia

## Abstract

**Background:**

Armed conflict can severely disrupt healthcare delivery, particularly in regions facing limited resources. There is little data on how the armed conflict in Tigray, Ethiopia, affected care for people with advanced HIV (AHD), especially those with cryptococcal meningitis (CM), making it hard to plan recovery efforts. This study explores the experiences of healthcare providers at Ayder Comprehensive Specialized Hospital in delivering care for AHD and CM before, during, and after the recent armed conflict in northern Ethiopia.

**Figure:**
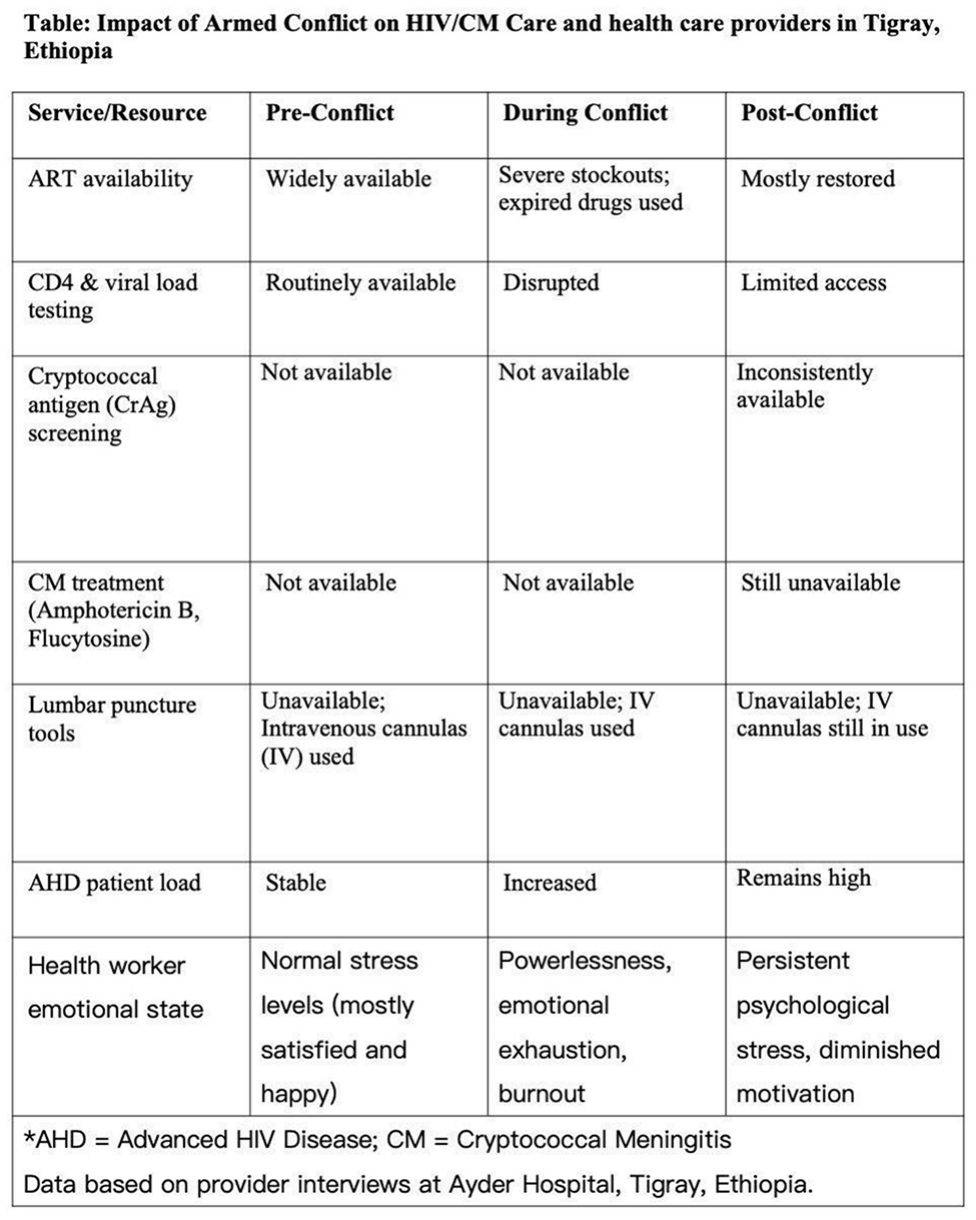

**Methods:**

We conducted in-depth interviews with neurologists, internists, medical residents, nurses, laboratory technicians, and pharmacists involved in AHD and CM care. About 13 Participants were purposely sampled to capture diverse experiences across different periods. Recruiting more participants continued until we reached theoretical saturation. The interviews were transcribed, translated, and thematically analyzed.

**Results:**

Health workers felt deeply hurt and powerless as patients died from preventable causes due to missing HIV and CM medicines. Many experienced burnout, lost empathy, and carried lasting emotional stress. Before the armed conflict, HIV services like antiretroviral therapy (ART), CD4, and viral load testing were widely available. The war caused major disruptions; ART stock outs led to the use of expired drugs, and basic lab tests stopped entirely. Lumbar punctures are still done with IV cannulas due to a lack of kits, leading to reluctance and failures. Cryptococcal Antigen screening was unavailable before the war and remains inconsistent. CM treatments like Amphotericin B and Flucytosine have never been accessible. Since the conflict, health workers report more patients presenting with AHD.

**Conclusion:**

The armed conflict had a devastating impact on both healthcare providers and the delivery of AHD and CM services. Providers endured profound emotional and professional strain, while critical shortages in medications and diagnostics compromised patient outcomes. These findings emphasize the urgent need for conflict-sensitive health system resilience strategies and targeted post-conflict recovery efforts.

**Disclosures:**

All Authors: No reported disclosures

